# Investigation of Storage Conditions and Quality Control Markers for Metabolites and Lipids in Human Feces

**DOI:** 10.3390/metabo16020113

**Published:** 2026-02-04

**Authors:** Hiroshi Sawada, Motohiko Morihara, Masamitsu Gotou, Kazuyuki Fujii, Yuya Hidoh, Yasuhiro Sawai, Takashi Matsumoto, Taiki Nakaya, Osamu Miura, Tomohiro Ando, Kazutaka Ikeda, Jun Terauchi

**Affiliations:** 1 Otsuka Pharmaceutical Co., Ltd., 463-10 Kagasuno, Kawauchi-cho, Tokushima, Tokushima 771-0192, Japan; fujii.kazuyuki@otsuka.jp (K.F.); hido.yuya@otsuka.jp (Y.H.); sawai.yasuhiro@otsuka.jp (Y.S.); 2 Japan Microbiome Consortium (JMBC), 3-1 Ofukacho, Kita-ku, Osaka, Osaka 530-0011, Japan; ma.gotou@ono-pharma.com (M.G.); matsumoto_takashi@mail.tsumura.co.jp (T.M.); nakaya_taiki@mail.tsumura.co.jp (T.N.); jun@metagentx.com (J.T.); 3 Ono Pharmaceutical Co., Ltd., 3-1-1, Sakurai, Shimamoto-cho, Mishima-gun, Osaka 618-8585, Japan; 4 TSUMURA & CO., 3586 Yoshiwara, Ami-machi, Inashiki-gun, Ibaraki 300-1192, Japan; 5 Axcelead Drug Discovery Partners, Inc., 26-1 Muraoka-Higashi 2-chome, Fujisawa, Kanagawa 251-0012, Japan; osamu.miura@axcelead.com (O.M.); tomo.and1981@gmail.com (T.A.); 6 Kazusa DNA Research Institute, 2-6-7 Kazusa-kamatari, Kisarazu, Chiba 292-0818, Japan; kaikeda@kazusa.or.jp; 7 Metagen Therapeutics, Inc., 1-6-1 Otemachi, Chiyoda-ku, Tokyo 100-8185, Japan

**Keywords:** human feces, quality control marker, metabolite, lipid, storage condition

## Abstract

**Background/Objectives**: The stability of metabolites and lipids in feces varies depending on the storage temperature and duration. **Methods**: We examined the stability of various metabolites and lipids in human feces under 10 different storage conditions (room temperature for 2, 6, 24, and 48 h, 4 °C for 6, 24, and 48 h, −20 °C for 1 week, 2 weeks and 1 month) and explored markers useful for quality control of fecal samples, using metabolites and lipids that vary depending on temperature and time. **Results**: There was generally more variation at 4 °C than at −20 °C, and more at room temperature than at 4 °C, and variation also increased as the storage duration was extended under each temperature condition. Some metabolites and lipids were found to be unstable, even over short periods (2 or 6 h) at room temperature or 4 °C storage. However, storage at −20 °C generally maintained the stability of most of them for up to two weeks. Our results suggest that the following ratios can serve as useful quality control markers: methionine to S-methyl-5-thioadenosine, xanthine to inosine and N-linoleoyl leucine to 1,2-dilinoleoyl-sn-glycerol. **Conclusions**: For comprehensive metabolite and lipid analysis, we recommend promptly transferring samples to −80 °C storage, except when stored at −20 °C for no longer than two weeks, with checks on markers for quality control. When measuring specific metabolites or lipids, our catalog data can be consulted to determine acceptable storage conditions.

## 1. Introduction

In recent years, increasing attention has been devoted to the microbiome, particularly gut bacteria. To promote the industrial application of microbiome research, the Japan Microbiome Consortium (JMBC) was established by Japanese industry in 2017. Currently, JMBC is working on establishing measurement standards within a reliable measurement infrastructure [1,2] and acquiring gut microbiome data from healthy individuals. Additionally, research is being conducted to obtain more valuable data by simultaneously measuring both the metabolome and the microbiome using human fecal samples. Because fecal metabolites, as well as the microbiome, can be sensitive to storage conditions, it is necessary to examine the stability of fecal samples under various storage conditions. To collect and preserve the metabolite and lipid components in fecal samples under the most stable conditions, it is generally recommended that samples should be stored immediately after collection in liquid nitrogen or in a freezer at −80 °C. While these conditions can usually be met in clinical trials where collection takes place at medical facilities, in many cohort studies, fecal samples are collected at home and stored in household freezers, refrigerators, or at room temperature [3,4]. When planning research or analyzing data for metabolome and lipidome analysis using fecal samples, it is essential to accurately understand the impact of differences in storage temperature and duration on metabolite component levels.

Several papers have reported on the stability of metabolites and lipids in feces. Spiegeleer et al. evaluated storage stability at −20 or −80 °C, but freeze-dried the feces before storage, stored them in the dried state, and evaluated the stability of metabolites [5]. Gratton et al. stored the feces without drying and evaluated stability at room temperature, in the refrigerator, frozen, and even after freezing and thawing [6]. Different metabolite classes exhibit different degrees of stability under different storage conditions [3,4,5]. New sampling kits, such as the OMNImet GUT kit, are being developed that allow for storage at room temperature [7,8]. However, there has been no identification of quality control metabolites to check for poorly stored samples. This study aims to catalog the impact of differences in storage temperature and duration on metabolite component levels and to identify quality control metabolites that allow exclusion of poorly stored samples. To achieve this, we measured metabolite component levels in fecal samples stored at room temperature, in a refrigerator, and in a freezer and compared them with samples stored at −80 °C.

## 2. Materials and Methods

### 2.1. Human Fecal Samples

The fecal samples, one from each of 8 healthy individuals (Caucasians) were obtained by Cantor BioConnect, Inc. (Santee, CA, USA) and purchased as 8 individual specimens from BizCom Japan, Inc. (Tokyo, Japan). The samples were stored at −80 °C immediately after collection (within 30 min) and transported in containers cooled by dry ice. After transportation, the fecal samples were stored at −80 °C until use. All samples were negative for HIV-1, HCV, and HBV.

### 2.2. Ethics Statement

All study protocols were approved by the Non-Profit Organization MINS Research Ethics Committee (Tokyo, Japan, Protocol number JMBC2023001).

### 2.3. Human Fecal Processing

Frozen fecal samples were pulverized and homogenized using a ShakeMaster AUTO (Bio Medical Science Inc., Tokyo, Japan). One sample was excluded from the study due to insufficient homogenization. To minimize individual variability for the stability test, the remaining seven samples were pooled in equal volumes. This pooled fecal sample was divided into aliquots and stored under the following temperature and storage duration conditions (*n* = 3 per condition): −80 °C; −20 °C for one week, two weeks, and one month; 4 °C for 6, 24, and 48 h; and room temperature for 2, 6, 24, and 48 h. The sample preserved at −80 °C was used as the reference (gold standard). Three aliquots of pooled samples were used for each storage condition, and each aliquot was measured once. Inter-individual variability was not assessed. Each fecal sample was mixed with methanol to achieve a final composition of 75 wt% methanol, followed by homogenization using a ShakeMaster AUTO. All extracts were immediately stored at −80 °C until metabolomic and lipidomic analysis.

### 2.4. Hydrophilic Metabolite HILIC/MS/MS Analysis

The 25% fecal methanol extracts were diluted 24-fold with methanol to prepare a 1% concentration. These diluted extracts were then mixed with 400 mM ammonium formate solution at a ratio of 19:1 (*v*/*v*), followed by vigorous vortexing and centrifugation at 21,500× *g* for 5 min at 4 °C. The resulting supernatant was analyzed using a Nexera UPLC system (Shimadzu Corporation, Kyoto, Japan) coupled with a QTRAP 5500 mass spectrometer (AB Sciex LLC, Marlborough, MA, USA). Metabolite separation was achieved on a ZIC-cHILIC column (2.1 mm I.D. × 100 mm, 3 μm, Merck Millipore, Darmstadt, Germany) maintained at 30 °C. The mobile phases consisted of (A) 10 mM ammonium formate in water and (B) acetonitrile, delivered at a flow rate of 0.4 mL/min using gradient elution. The gradient program was as follows: 0–1.5 min, 97% B;1.5–5 min, 97–75% B;5–7 min, 75% B; 7–10 min, 75–40% B;10–12 min, 40% B; 12–13 min, 40–10% B; 13–16 min, 10% B;16–25 min, 97% B.

The eluent was ionized via electrospray ionization (ESI) and monitored with multiple reaction monitoring (MRM) mode, as described previously [9]. The MRM data were processed using MultiQuant 3.0 (AB Sciex Pty). Metabolites were identified by comparing their retention times and MRM transition with those of authentic standard compounds.

### 2.5. Hydrophilic Sulfate Metabolite Analysis

For the analysis of biological thiols, the 25% fecal methanol extracts were diluted 24-fold with methanol to a final concentration of 1%. Thiol groups were then alkylated by adding 0.1 volumes of 20 mM β-(4-hydroxyphenyl)ethyl iodoacetamide (HPE-IAM), followed by incubation for 1 h at 37 °C. The reaction mixture was centrifuged at 21,500× *g* for 5 min at 4 °C. The resulting supernatant was mixed with 400 mM ammonium formate at a ratio of 19:1 (*v*/*v*), vortexed, and centrifuged again at the same conditions. The final supernatant was analyzed by the same procedure used for HILIC/MS/MS analysis described above.

### 2.6. Hydrophilic Metabolite GC/MS/MS Analysis

A 10% methanol extract was prepared by adding 1.5 volumes of methanol to the initial 25% extract. The mixture was centrifuged at 21,500× *g* for 5 min at 4 °C. Stable isotope-labeled internal standards were added to the resulting supernatant, which was then evaporated to dryness under a gentle stream of nitrogen gas. The dried residues were derivatized via oxidation followed by trimethylsilylation. The derivatized metabolites were analyzed using an Agilent 7890B gas chromatography system coupled with an Agilent 7010 series triple-quadrupole mass spectrometer (Agilent Technologies Inc. Santa Clara, CA, USA). Chromatographic separation was achieved on a DB-5MS column (30 m × 0.25 mm i.d., df = 0.25 μm, Agilent Technologies). Helium was used as the carrier gas with a temperature gradient from 60 °C to 325 °C at 10 °C/min. The effluent was ionized via electron impact (EI) and analyzed in MRM mode. The MRM peak area was calculated using MassHunter software version B.07.00 (Agilent Technologies Inc.).

### 2.7. Unbiased Lipidomics Method

Unbiased lipidomics was performed as described previously with some modifications [10,11]. Briefly, 10 µL of the methanol suspension of human fecal samples was mixed with 300 µL of chloroform:methanol:water (1:2:0.2) containing EquiSPLASH (Avanti Polar Lipids, Alabaster, AL, USA) for internal standards. After sonication for 30 s, the solutions were vigorously agitated at 750 rpm for 40 min at 20 °C. The mixture was centrifuged at 1670 g for 10 min at 20 °C and the supernatant was transferred to an LC vial. LC/MS/MS analysis was carried out using quadruple time-of-flight (Q TOF)/MS (TripleTOF 6600; AB Sciex LLC, Framingham, MA, USA) coupled with an ACQUITY UPLC system (Waters Corporation, Milford, MA, USA).

The LC separation was performed with gradient elution of mobile phase A [methanol/acetonitrile/water (1:1:3, *v*/*v*/*v*) containing 5 mM ammonium acetate (FUJIFILM Wako Chemicals, Osaka, Japan) and 10 nM EDTA (Dojindo, Japan)] and mobile phase B [isopropanol (Wako Chemicals) containing 5 mM ammonium acetate and 10 nM EDTA]. The flow rate was 300 µL/min at 45 °C using an L-column3 C18 (50 × 2.0 mm i.d., particle size 2.0 µm; Chemicals Evaluation and Research Institute). The gradient program was as follows: 0 min, 0% (B); 6.5 min, 64% (B); 13.5 min, 76.5% (B); 18 min, 98% (B); 20 min, 98% (B); 20.1 min, 0% (B); and 25 min, 0% (B).

MS analysis was performed in high-resolution mode for MS1 (~37,385 full width at half maximum, FWHM) and in high-sensitivity mode for MS2 (~29,474 FWHM) using data-dependent acquisition (DDA). The MS parameters were as follows: MS1 scan range, *m*/*z* 140–1700; MS2 scan range, *m*/*z* 75–1700; MS1 accumulation time, 250 ms; MS2 accumulation time, 100 ms; collision energy, +45/–42 eV; collision energy spread, 15 eV; cycle time, 1301 ms; curtain gas, 30; ion source gas 1, 40 (+)/42 (–); ion source gas 2, 80 (+)/50 (–); temperature, 250 °C (+)/300 °C (–); ion spray voltage floating, +5.5/–4.5 kV; and declustering potential, 80 V.

The raw data files were converted to MGF format using the Sciex MS Data Converter software version V1.3 beta and subjected to quantitative analysis with 2DICAL (Mitsui Knowledge Industry, Tokyo, Japan). Lipid molecular species were identified using MS1 exact mass, retention times and MS/MS spectra acquired under DDA conditions, considering MS/MS fragment peak weighting and similarity.

### 2.8. Statistical Analysis

Each condition was repeated three times for the experiment. For each analysis, the value was normalized by dividing it by the average value at −80 °C, and the log_2_-transformed value was calculated. Multiple comparisons were performed using Dunnett’s method implemented in software R (version 4.5.1) with the package ‘multcomp’. Although Dunnett’s test was applied to account for multiplicity across multiple evaluation points, the resulting *p*-values were not adjusted for multiplicity across multiple metabolites. In such cases, the *p*-values are therefore reported as nominal *p*-values. Metabolites with *p*-values less than 0.05 and a fold change greater than 2 were considered increased, whereas those with *p*-values less than 0.05 and a fold change less than 0.5 were considered decreased.

## 3. Results

Global metabolomics using HILIC/MS/MS, GC/MS/MS, and LC/MS/MS detected 241 metabolites and 777 lipids in human feces (Supplemental Tables S1 and S2).

Among 241 hydrophilic metabolites, metabolites of the Cys/Met pathway were the most abundant, followed by metabolites of the purine pathway and metabolites of the bile acid pathway (Table 1). Neutral glycerolipids, such as triglycerides, accounted for the highest percentage among the 777 identified lipids. A large number of glycerophospholipids, sterol esters, fatty acids, and bile acid esters were also detected (Table 2).

**Figure 1:**
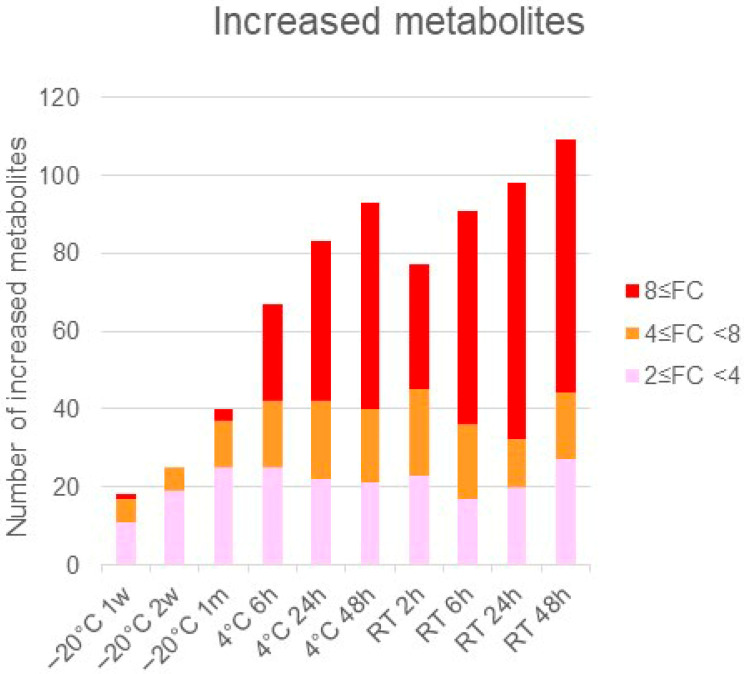

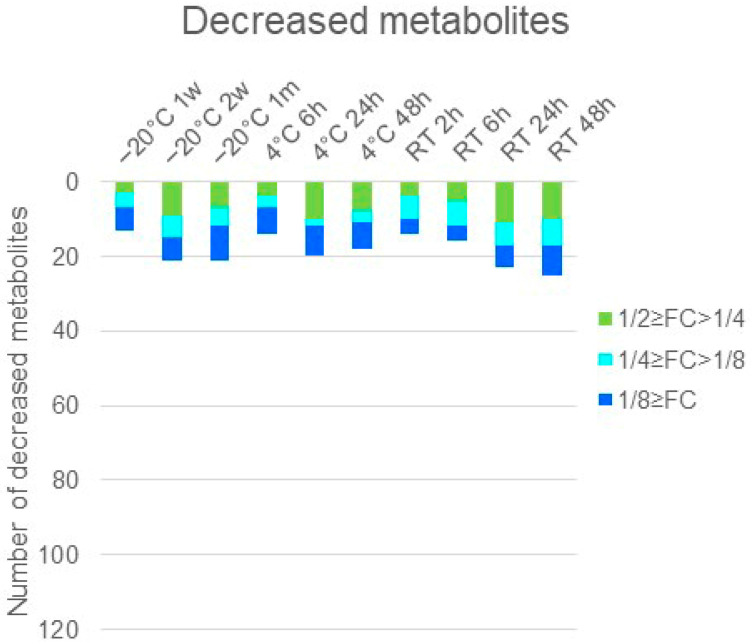
Temperature- and time-dependent changes in metabolite quantity: the values obtained following storage at −80 °C were considered control values, and those obtained under each condition were compared with the control. The metabolites with *p*-values less than 0.05 and a fold change greater than 2 or less than 0.5 were counted under each condition, and the number was highlighted according to the fold change (FC) ratio.

**Figure 2:**
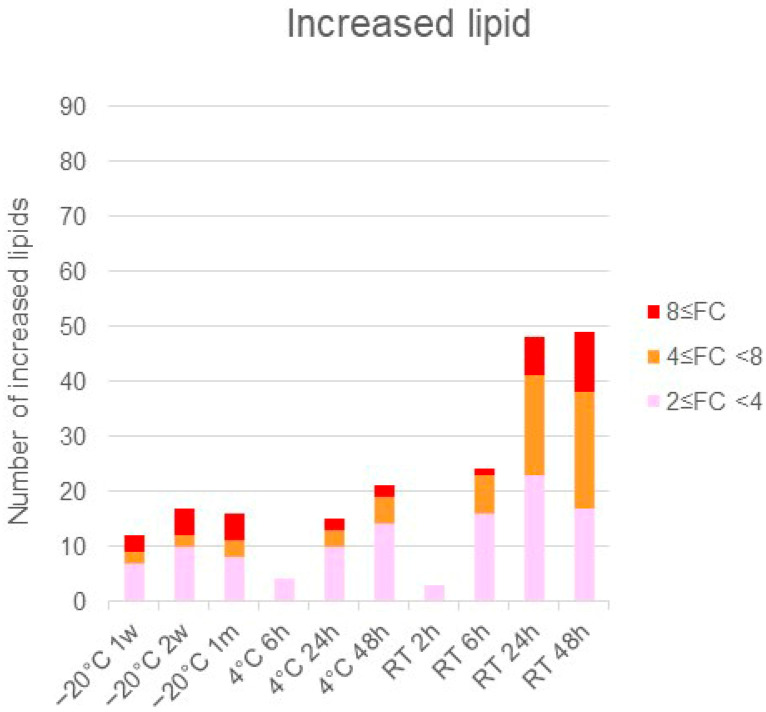

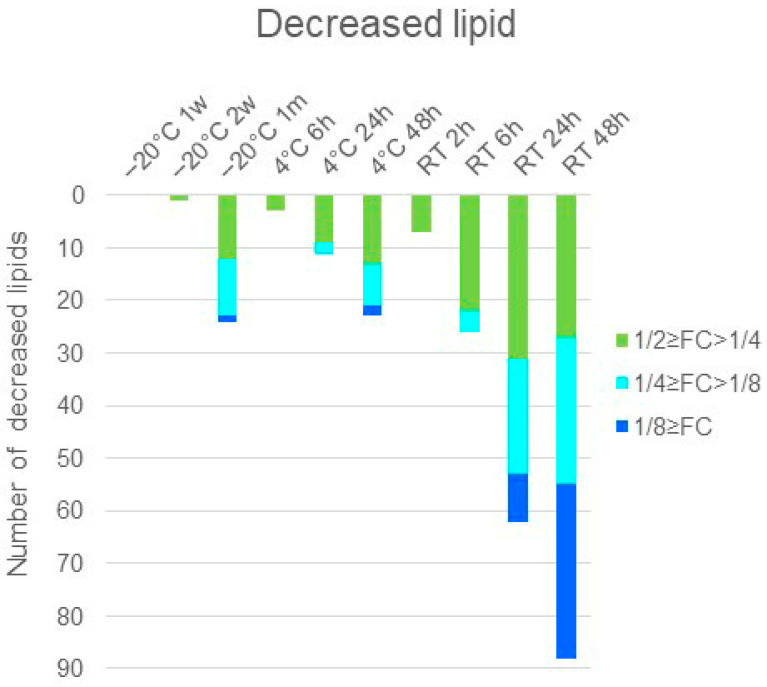
Temperature- and time-dependent changes in lipid quantity; the values obtained following storage at −80 °C were considered control values, and those obtained under each condition were compared with the control. The lipids with *p*-values less than 0.05 and a fold change greater than 2 or less than 0.5 were counted under each condition, and the number was highlighted according to the fold change (FC) ratio.

The significantly altered metabolites (*p* < 0.05 and fold change >2 or <0.5) under each condition were identified by comparison with the values obtained following storage at −80 °C (Supplemental Tables S3 and S4). The number of changed metabolites and lipids depended on greater excursions of temperature and time (Figure 1 and Figure 2).

The quantity of metabolites varied depending on the storage temperature conditions and duration. There were generally more changes at 4 °C than at −20 °C, and even greater changes at room temperature than at 4 °C. These changes increased progressively with longer storage duration under each temperature condition. Up to 2 weeks of storage at −20 °C, the total number of metabolites showing either an increase or a decrease remained below 50, suggesting that the differences from samples stored at −80 °C were relatively minor. Conversely, under other temperature and storage durations, where variation becomes greater, we recommend changing from storage at −20 °C to −80 °C within 2 weeks when storing samples for longer. The variation in components was observed to have trends that depended on the metabolite categories.

**Figure 3:**
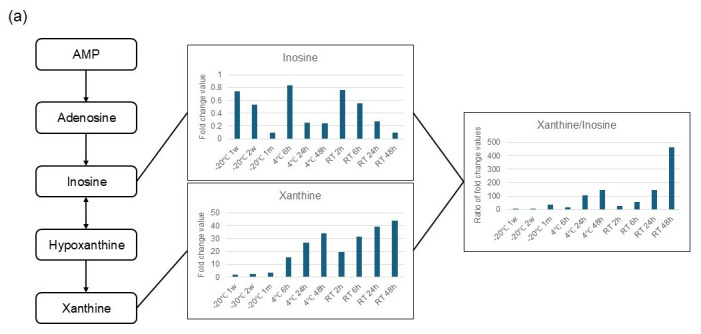

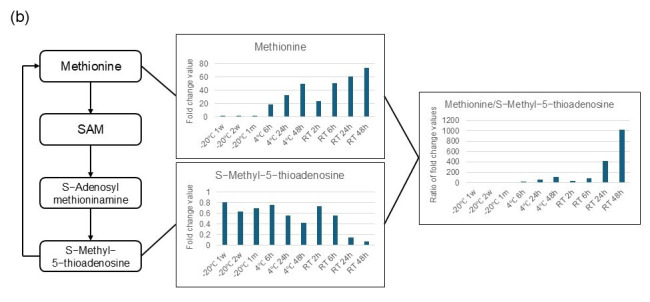
The quality control markers (ratio of xanthine to inosine (**a**) and methionine to S-methyl-5-thioadenosine (**b**)) reflect metabolic stability under each storage condition. The y-axis shows the fold change relative to the −80 °C storage condition for each storage condition. Three aliquots of pooled samples were measured for each storage condition.

Markers for evaluating or excluding changes caused by improper handling or storage conditions are crucial for the quality control of fecal metabolomics samples but have not been reported to date. Significant, time-dependent fluctuations were also observed in metabolites related to purine metabolism. Adenosine and inosine significantly decreased over time, whereas hypoxanthine and xanthine increased. This suggests that the degradation of ribonucleic acid and deoxyribonucleic acid under long-term storage at room temperature or refrigerated conditions led to an increase in the downstream metabolites hypoxanthine and xanthine. In addition, Chen et al. investigated changes in viable microbiota at the phylum and genus levels in relation to fecal storage temperature and reported that the proportion of Pseudomonadota (Proteobacteria) significantly increased when samples were stored at room temperature [12]. Pseudomonadota possess diverse metabolic capabilities and are deeply involved in purine metabolism [13]. These findings suggest that the xanthine value or the xanthine-to-inosine ratio (Figure 3a) would be useful markers to assess the quality control of samples.

Significant fluctuations were also observed in the Cys/Met pathway. Methionine increased over time under room temperature and refrigerated conditions, while S-methyl-5-thioadenosine decreased. Additionally, neither compound showed significant fluctuations when stored at −20 °C. Although the metabolic reaction mechanism is unknown, the change in methionine is consistent with the report by Gratton et al. [6], and obtaining the ratio of these compounds (methionine/S-methyl-5-thioadenosine) may serve as a marker to evaluate the stability of storage after sample collection (Figure 3b).

**Figure 4:**
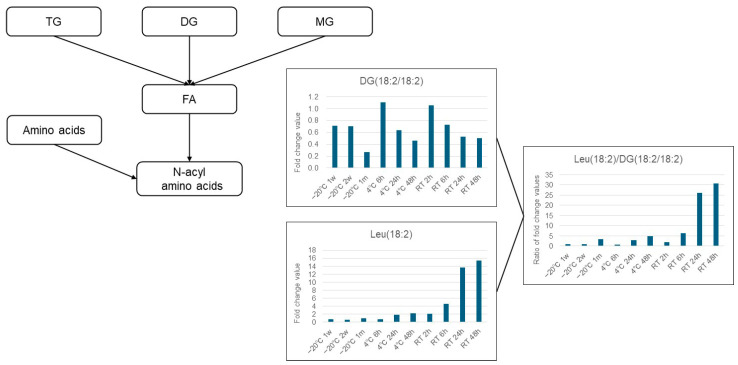
The quality control marker (ratio of Leu (18:2) to DG (18:2/18:2)) reflects lipidomic stability under each storage condition. The y-axis shows the fold change relative to the −80 °C storage condition for each storage condition. Leu: leucine, DG: diglyceride. Three aliquots of pooled samples were measured for each storage condition.

We explored quality control markers that could serve as indicators of stability for lipids. Focusing on metabolites exhibiting time-dependent fluctuations, N-linoleoyl leucine (Leu (18:2)) increased over time under both room temperature and refrigerated conditions, while DG (18:2/18:2) (1,2-dilinoleoyl-sn-glycerol) showed a decreasing trend. The enzyme aminoacylase, which is involved in the reaction between amino acids and fatty acids, can be produced by a wide range of bacteria [14]. It is possible that the increase in acyl amino acids (e.g., Leu (18:2)) over time during room temperature and refrigerated storage is influenced by residual aminoacylase in feces (Figure 4). Thus, the increase in the ratio of Leu (18:2) to DG (18:2/18:2) was suggested to be useful as a stability indicator for fecal samples in lipidomics analysis (Figure 4).

By using these variations as indicators, it may be possible to evaluate whether appropriate sample handling and extraction procedures were performed and whether storage under frozen conditions was maintained. The findings are exploratory, and further validation of their utility as a marker will require studies with an increased sample size.

## 4. Discussion

Compared to samples stored at −80 °C, various changes in metabolites were observed under each storage condition, with the largest changes seen in relation to water-soluble metabolites such as amino acids, sugars, and nucleic acids (see Table S3).

Although some metabolites like arginine decreased, most amino acids increased with increasing temperature and duration. It has been reported that proteins and proteases are present in feces, and under certain conditions, protein concentrations decrease during storage [15]. Additionally, the action of bacteria-specific trypsin has also been reported [16]. These findings suggest that proteins and other sources of amino acids in feces are broken down by proteases during storage, leading to an increase in amino acid levels. Previous studies evaluating the stability of metabolites in feces [5,6] have shown that amino acid fluctuations are not uniform, indicating that differences in substances contained in the evaluated feces (e.g., residues of food) and fecal processing conditions (e.g., presence or absence of freeze-drying) may result in varying changes in amino acids.

When observing sugars, disaccharides and trisaccharides tended to decrease, while monosaccharides tended to increase compared to storage at −80 °C. It is known that feces contain enzymes such as glucosidases that hydrolyze the bonds between sugars [17], and the opposing changes in metabolites bound to sugars and monosaccharides can be attributed to the influence of these enzymes. The increase in monosaccharides was dependent on time and storage temperature. On the other hand, the decreased amounts of disaccharides like maltose and trisaccharides like maltotriose did not correlate with storage duration or temperature. Disaccharides and trisaccharides may not only disappear through metabolism but may also be generated from larger polysaccharides, suggesting that under certain conditions, their production and degradation were balanced.

Purine-related metabolites also varied with storage, with hypoxanthine and xanthine increasing under all temperature conditions. Purines and pyrimidines are molecules necessary for the synthesis of nucleic acids but are also produced by the decomposition of nucleic acids. A study in which DNA was extracted from feces and measured showed that DNA bands disappeared in a time- and temperature-dependent manner [18]. Another study also reported that DNA and RNA fragmentation occurred when fecal samples were stored at room temperature [19]. Since feces contain bacteria and host-derived cells, it is possible that the DNA and RNA in the cells were damaged depending on the storage duration and temperature, resulting in an increase in hypoxanthine and xanthine. In other words, when measuring nucleotide-related metabolites and purine-related metabolites in feces, care must be taken when handling the feces, such as storing the samples at ultra-low temperatures to prevent the activity of DNase and RNase in the samples, extracting with organic solvents, or adding inhibitors of these enzymes to prevent nucleic acid damage. On the other hand, adenosine decreased regardless of storage duration or temperature. Since some amino acids and sugars change even at −20 °C, it is possible that some cellular functions and enzyme activity remain even at low temperatures, and adenosine, which is involved in energy metabolism, may have been used and decreased.

When storing samples for long-term analysis of lipid metabolites in feces, we recommend storage at −80 °C as soon as possible. Even at room temperature for 2 h, there are components that vary, so future studies must take care in handling samples and interpreting data when evaluating the lipid variation in human feces. When stored at −20 °C for one week or two weeks, 17 lipids were increased and 1 lipid) was decreased, but there was no change in other lipids. When storing fecal samples containing lipids at −20 °C, we recommend transferring them to a −80 °C freezer within two weeks for long-term storage. When stored at −20 °C for one month, 24 lipids decreased and 16 lipids increased. Among the lipids that decreased, many were triglycerides. The tendency for TGs to decrease suggests TGs may have been decomposed by lipase. An increase was observed in wax esters and LPEt (18:1). When stored at 4 °C for 6 h, four types of lipids increased and three types of lipids decreased, but there was no change in other lipids. When stored for 24 and 48 h, many wax esters increased, and some N-acyl amino acids, glycerolipids, fatty acids, glycerophospholipids and sterol esters increased. Conversely, glycerolipids (lysophospholipids) and glycerophospholipids decreased. When storing samples for long-term lipid metabolite analysis in feces, we recommend storing them at −80 °C within 6 h, if currently stored at 4 °C. When stored at room temperature for 2 h, 3 lipids increased and 7 lipids decreased. The components that decreased were mostly lysophospholipids, and we recommend storing them at −80 °C as soon as possible after stool collection when evaluating these lipid metabolites. When stored at room temperature for 48 h, many lipids such as wax esters, N-acyl amino acids, glycerolipids, bile acids, sterol esters, and neutral glycerolipids increased, while many lipids such as glycerolipids decreased (see Table S4).

**Figure 5:**
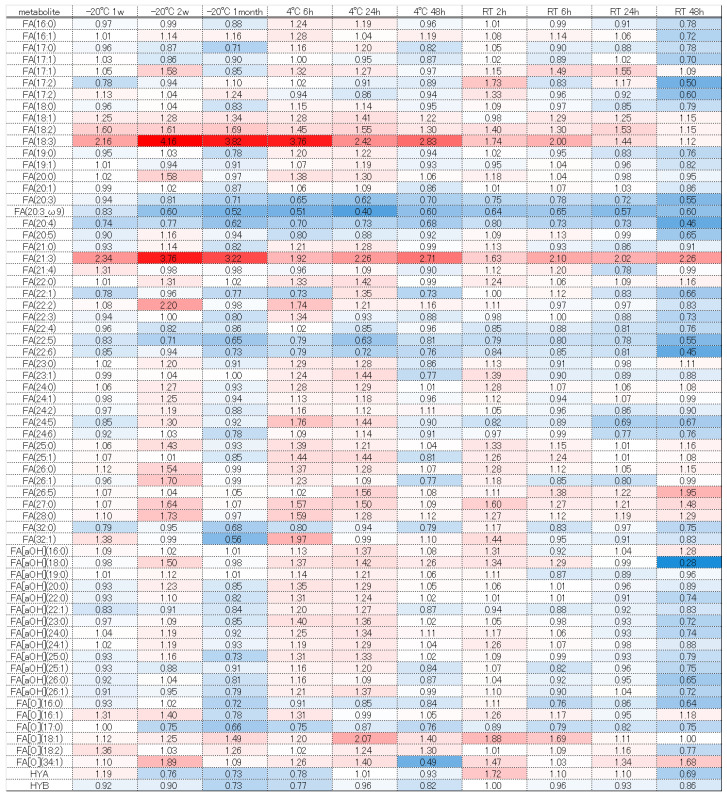
Heatmap of quantitative fluctuations in the group of free fatty acids due to temperature conditions and storage duration. Components that increased compared to storage at −80 °C are indicated with a red label, while those that decreased are indicated with a blue label.

**Figure 6:**
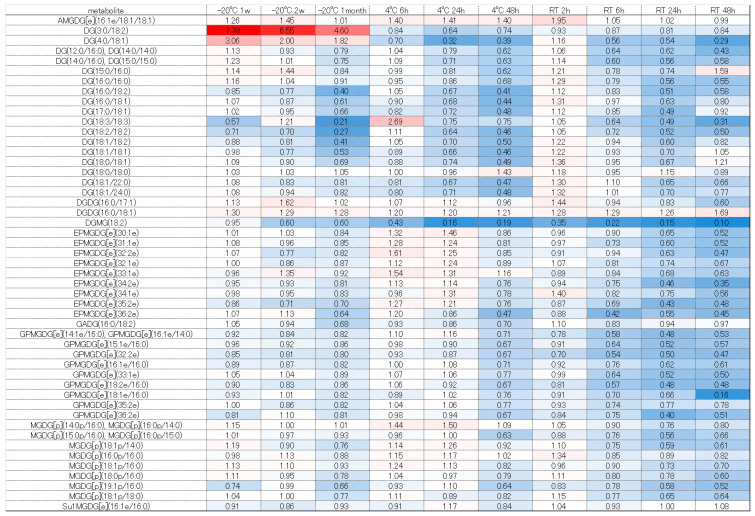
Heatmap of quantitative fluctuations in the group of diacylglycerols due to temperature conditions and storage duration. Components that increased compared to storage at −80 °C are indicated with a red label, while those that decreased are indicated with a blue label.

**Figure 7:**
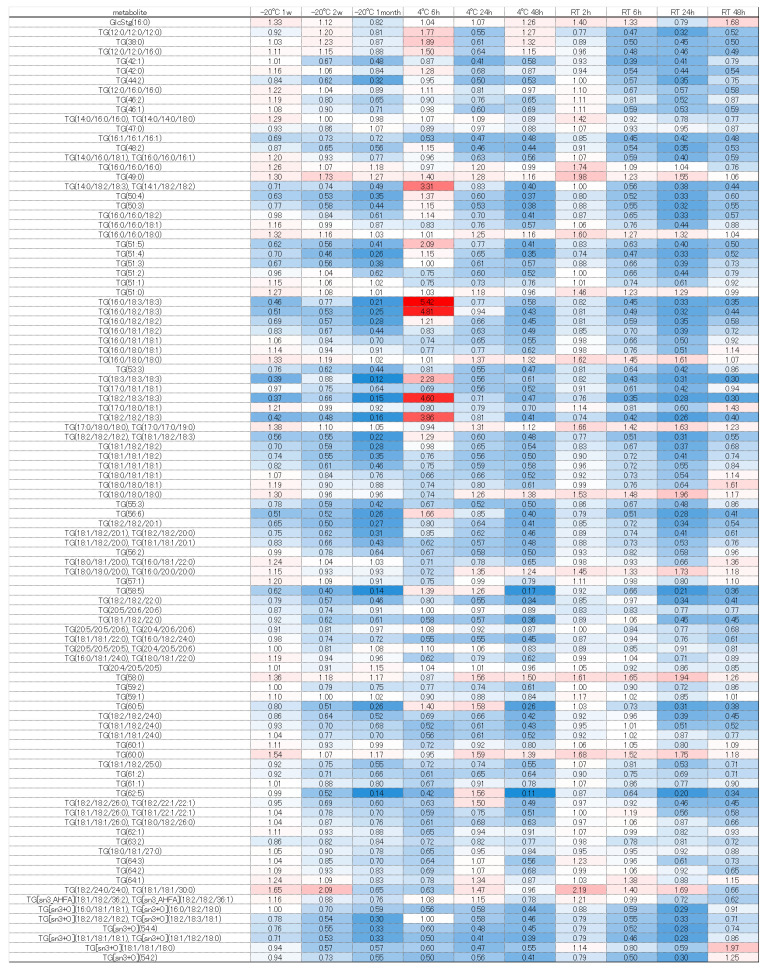
Heatmap of quantitative fluctuations in the group of triacylglycerols due to temperature conditions and storage duration. Components that increased compared to storage at −80 °C are indicated with a red label, while those that decreased are indicated with a blue label.

**Figure 8:**
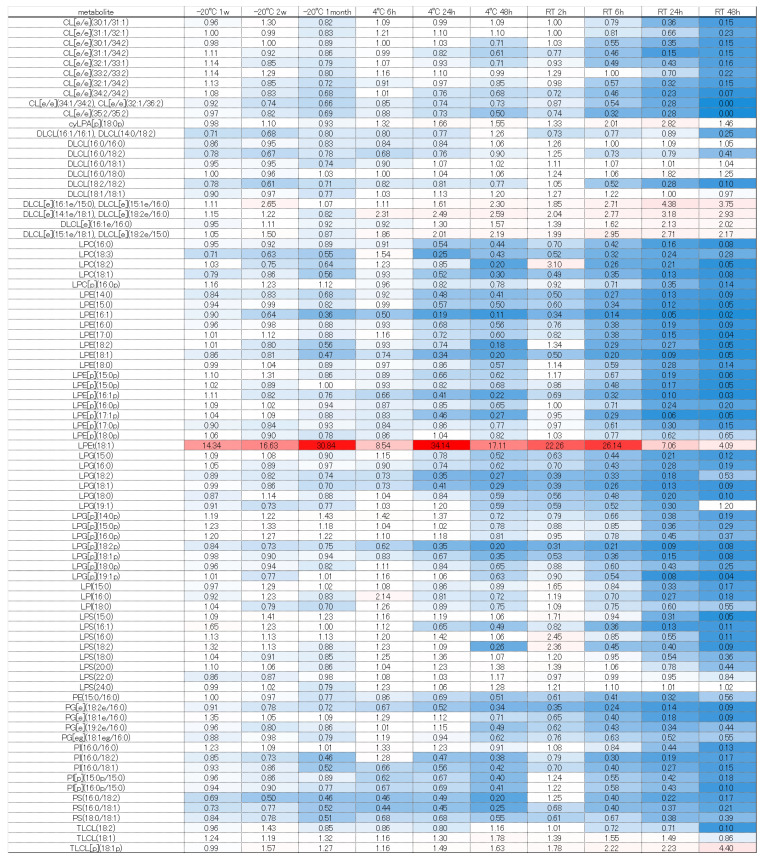
Heatmap of quantitative fluctuations in the group of glycerophospholipids due to temperature conditions and storage duration. Components that increased compared to storage at −80 °C are indicated with a red label, while those that decreased are indicated with a blue label.

**Figure 9:**
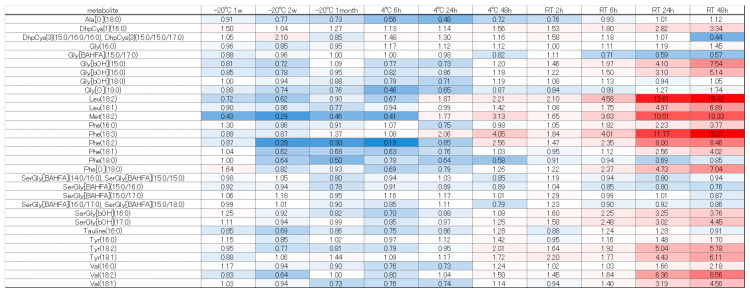
Heatmap of quantitative fluctuations in the group of N-acyl amino acids due to temperature conditions and storage duration. Components that increased compared to storage at −80 °C are indicated with a red label, while those that decreased are indicated with a blue label.

Next, we considered the trends of each component group using a heatmap comparing the fold change, with −80 °C as the gold standard. Free fatty acids such as FA (18:2), (18:3), and (21:3) tended to increase, which may have been due to lipase degradation from DG, TG, and others (Figure 5). Conversely, many fatty acids decreased at room temperature when stored for 48 h. Under storage at room temperature, even free fatty acids may decompose and decrease, or they may be used for increasing components due to esterification, amidation, etc. However, further verification is necessary. Diacylglycerols and tricylglycerols tended to decrease overall as the storage duration was extended. These findings are probably attributable to progressive decomposition by lipase (Figure 6 and Figure 7). A large number of glycerolipids decreased; they are therefore easily decomposable and need to be stored quickly in a −80 °C freezer (Figure 8). N-acyl amino acids consist of molecules where the carboxyl group of fatty acids is bonded with amino acids such as glycine, serine, or ornithine through amide linkage. While there is an overall decline at −20 °C or 4 °C, an increase was noted at room temperature (Figure 9). We believe that under room temperature, the quantities of N-acyl amino acids increase due to the reactivation of gut bacteria or enzymes that promote amidation.

## 5. Conclusions

We have collected catalog data on the stability of various metabolites and lipids in feces. There was generally greater variation at 4 °C than at −20 °C, and more variation at room temperature than at 4 °C, and variation also increased as the storage duration extended under each temperature condition. Storage at −20 °C generally provided stable results for up to two weeks.

We explored markers for the quality control of fecal samples from metabolites and lipids that vary depending on temperature and time. The results suggest that the ratios of methionine to S-methyl-5-thioadenosine, xanthine to inosine, and Leu (18:2) to DG (18:2/18:2) represent novel marker candidates and are useful, although further verification is needed.

For comprehensive metabolite and lipid analysis, we recommend storage at −80 °C or at −20 °C for no more than two weeks and checking of markers for quality control. In measuring specific metabolites or lipids, these catalog data can be referred to when searching for acceptable storage conditions.

## Figures and Tables

**Table 1:** Number of metabolites in each category detected by using HILIC/MS/MS and GC/MS/MS.

Category	Number
Ala/Asp/Glu pathway	7
Aminobenzoate pathway	3
Arg/Pro pathway	10
BCAA pathway	12
Bile acid pathway	13
Cholesterol	2
Cys/Met pathway	21
Dicarboxylic acid	10
Fatty acyl carnitines	5
Free fatty acid	6
Glucose pathway	2
Gly/Ser/Thr pathway	9
Glycolysis	3
His pathway	4
Lipid pathway	6
Lys pathway	3
N-acetyl-amino acid	9
Nicotinate and nicotinamide pathway	3
Phe pathway	6
PPP	3
Purine pathway	14
Pyrimidine pathway	12
Steroid	2
Sugar related	11
TCA cycle	5
Trp pathway	8
Tyr pathway	9
Ubiquinone pathway	3
Vitamin	7
Others	33
Total	241

**Table 2:** Number of lipids in each category detected by using LC/MS/MS.

Category	Number
Acyl steryl glucoside	6
Acylcarnitine	6
Anandamide related	7
Bile acid ester	58
Estolides	16
Fatty acid	66
Ganglioside	10
Glyceroglycolipid	33
Glycerolipid	127
Glycerophospholipids	89
Glycosphingolipid	44
MIPC/GIPC	28
N-acyl amino acid	25
Phytyl ester	2
Prenol ester	3
Sphingo lipid	3
Sphingoid base	5
Sphingolipid	42
Sphingophospholipid	27
Sterol ester	89
Sterol metabolite	23
Wax ester	40
Others	28
Total	777

## Data Availability

The metabolomics data is deposited in the metabolomics workbench with datatrack_id:6832 and study_id:ST004598.

## Supplementary Materials

The following supporting information can be downloaded at: https://www.mdpi.com/article/10.3390/metabo16020113/s1, Table S1: List of 241 metabolites and the fold change values with −80 °C as the gold standard. Table S2: List of 777 lipids and the fold change values with −80 °C as the gold standard. Table S3: List of significantly changed metabolites with *p*-values less than 0.05 and fold change greater than 2 or less than 0.5. Table S4: List of significantly changed lipids with *p*-values less than 0.05 and fold change greater than 2 or less than 0.5.

## Abbreviations

The following abbreviations are used in this manuscript:

TG	Triglycerides
DG	Diglycerides
MG	Monoglycerides
JMBC	Japan Microbiome Consortium
HILIC	Hydrophilic Interaction Chromatography
MRM	Multiple Reaction Monitoring
GC	Gas Chromatography
LC	Liquid Chromatograph
FC	Fold Change
TCA	Tricarboxylic Acid
RT	Room Temperature
PPP	Pentose Phosphate Pathway
MIPC/GIPC	Mannosylinositolphosphoceramide/Glycosylinositolphosphoceramide
Leu (18:2)	N-Linoleoyl Leucine
DG (18:2/18:2)	1,2-Dilinoleoyl-sn-glycerol
